# Analysis of psychiatrists’ internet service patterns: a cross-sectional study from China’s largest online mental health platform

**DOI:** 10.3389/fpsyt.2025.1598574

**Published:** 2025-06-26

**Authors:** Tiannan Xu, Ruimei Ni, Hongye Wu, Feng Xu, Suqi Song, Xiaoping Yuan, Kai Zhang

**Affiliations:** ^1^ Department of Psychiatry, School of Mental Health and Psychological Sciences, Anhui Medical University, Hefei, China; ^2^ Department of Psychiatry, The Fourth Affiliated Hospital of Anhui Medical University, Hefei, China; ^3^ Haoxinqing Health Industry Group Co., Ltd., Beijing, China; ^4^ Anhui Psychiatric Center, Anhui Medical University, Hefei, China

**Keywords:** eHealth, internet hospital, online mental health care services, digital health, mHealth

## Abstract

**Background:**

Haoxinqing, China’s largest online mental health platform, facilitates digital psychological care delivery. This study aims to describe the demographics and medical service data of doctors on the Haoxinqing platform and investigate their associations.

**Method:**

The study analyzed the demographic information and medical service data of 11,333 registered physician users on the Haoxinqing platform over a 5-year period.

**Result:**

Among registered physicians, 87.0% were from secondary or tertiary hospitals and were concentrated in eastern provinces (e.g., Guangdong: 918). Female physicians had a lower proportion in senior titles (chief physicians: 19.0% vs. 20.0% for males), although the chi-square analysis indicated a weak association between gender and professional title (Cramer’s *V* = 0.051, *P* < 0.001). Text and image consultations dominate (82.1%). Professional titles significantly impacted service volume: chief physicians had 3.85 times more patients (IRR = 3.85, 95% CI [2.11–7.00]) and prescribed 4.16 times more medications (IRR = 4.16, 95% CI [3.21–5.41]) than residents (*P* < 0.001). Negative binomial regression showed that male physicians had 30% fewer patients than females (IRR = 0.70, 95% CI [0.58–0.85], *P* < 0.001), but the effect size for the association between gender and consultation methods was low (Cramer’s *V* = 0.036).

**Conclusion:**

Based on cross-sectional data from China’s largest online mental health platform, this study revealed that online services, while supplementing offline medical care, are still influenced by traditional medical hierarchy. Patients’ trust in senior physicians and gendered communication norms are critical determinants affecting resource allocation patterns on digital platforms.

## Introduction

Mental disorders remain one of the top ten global burdens (1). According to the World Mental Health Report 2022, in 2019, 970 million people worldwide suffered from mental disorders, which means that one in every eight people may have a mental disorder (2). In China, a mental health survey shows that the lifetime prevalence rate of mental disorders (excluding dementia) is 16.6%, implying that nearly 230 million Chinese people will suffer from mental illness in their lifetime (3).

However, mental health service resources in China are relatively limited. According to statistics from the National Health Commission, as of the end of 2021, the number of psychiatrists in China reached 64,000, accounting for only 1.49% of the total number of physicians nationwide (4.287 million) (4). The number of psychiatrists per 100,000 population is 3.64, which is still a significant gap compared to the 7.9 psychiatrists per 100,000 population in high - income countries (5). In addition, the geographical distribution of mental health resources in China is also uneven. The number of open psychiatric beds, physicians, and nurses per unit area of land in the western region is about 4 times less than that in the central region and 7–11 times less than that in the eastern region (6).

Since the 1950s, telepsychiatry has pioneered geographically unrestricted mental healthcare delivery through real-time videoconferencing technology. Early models relied on dedicated institutional networks, which mitigated geographic barriers but faced challenges including technical complexity and high patient access costs (7). The proliferation of mobile internet catalyzed the emergence of internet hospitals as a streamlined telemedicine modality, leveraging smartphone platforms to directly connect specialized medical resources with individual patients, thereby dismantling institutional barriers inherent to traditional remote care systems (8).

Internet hospitals have developed rapidly in China. As of June 2024, the number of Internet medical users in China has reached 365 million, accounting for 33.2% of the total number of Internet users (9). Since the Guangdong Provincial Second People’s Hospital established the first Internet hospital in China on October 25, 2014 (10), by September 12, 2024, according to the news release from the National Health Commission, the number of Internet hospitals in the country has reached 3,340, and the annual volume of Internet medical services provided exceeds 100 million visits (11). As China’s largest online mental health service provider, Haoxinqing collaborates with over 60,000 registered healthcare professionals, including 50,000 psychiatrists and psychologists, covering 80% of public hospitals nationwide (12).

Prior research has predominantly concentrated on patient needs, technological applications, and macro-level policies in internet hospitals, with less emphasis on the influence of individual physician behaviors on service models. From the patient perspective, Liu et al. (13) identified that trust and educational level significantly impact patients’ intentions to select platforms operated by different entities (13). Yu et al. (14) focused on shared decision-making between physicians and patients, highlighting the value of patient involvement in the formulation of online treatment plans (14). In terms of technology and policy, Shi et al. (15) explored the closed-loop management application of artificial intelligence in pediatric epidemic prevention and control (15). Han et al. (16) reviewed the industry’s development from a policy perspective, emphasizing the necessity of coordination between government regulation and market-oriented operations (16). Although Li et al. (17) analyzed the online consultation fee model with reference to physician service characteristics, the impact of professional titles and gender differences was not thoroughly examined (17).

Demographic attributes of physicians—particularly gender, age, and professional rank—serve as pivotal variables in dissecting healthcare service disparities. Grounded in Rogers’ Diffusion of Innovations theory, technology adoption follows an S-shaped curve segmented into innovators, early adopters, early majority, late majority, and laggards. Professional hierarchy and age may determine physicians’ positioning within this adoption continuum (18). For instance, senior clinicians often act as early adopters due to their academic authority and institutional influence. Empirical evidence supports this: Gong et al. (19) demonstrated a significant positive correlation between physicians’ professional rank (proxied by seniority) and online consultation volume (*β* = 0.815, *P* < 0.01), indicating that higher-ranked physicians attract greater patient trust and engagement (19).

Social role theory further reveals that society has preset behavioral norms for different genders (i.e., “role expectations”), which are conveyed through culture, institutions, and interpersonal interactions, influencing individuals’ behavioral patterns and evaluation criteria in their professions (20). Gender expectations shape medical behavior. Studies have shown that women need to meet the “dual role expectations” of both professional competence and emotional care, while men’s deficiencies in soft skills such as interpersonal communication and emotional interaction are more easily tolerated (21).

In summary, the impact of physician demographic characteristics on medical service data permeates dimensions such as resource allocation and physician - patient interaction, holding both theoretical value in revealing patterns of medical services and practical significance in optimizing medical resource allocation and improving service quality. This study aims to: 1) characterize the demographic profile of physicians registered on the Haoxinqing platform; 2) delineate patterns in telemedicine service data; 3) analyze how physicians’ demographic attributes influence service delivery outcomes.

## Method

### Data collection and ethical considerations

We collected comprehensive data from the Haoxinqing Internet Hospital platform from January 2018 to June 2023. The dataset mainly includes demographic data of registered mental health professionals and medical service data generated during this period. The demographic characteristics are detailed to include gender distribution, average age, as well as the professional titles and number of hospitals involved. Medical service data includes the number of patients who established a relationship with the doctors, the number of prescriptions issued, and the total number of consultations, which can be further classified into text-and-image consultations, telephone consultations, and video consultations. **Table 1** lists the relevant data related to this study.

**Table 1:** The statistical measure designed in this study.

Variable	Value
Demographic characteristics	
Total registered mental healthcare professionals, N	11333
Gender (M/F)	5659/5674
Age, mean (SD)	43.81(9.69)
Number of professional titles, n	16
Number of hospitals, n	2532
Medical service data	
Volume of patients formed relations, (M/F)	1402366/953721
Volume of prescriptions, (M/F)	907254/754806
Total consultation, (M/F)	293720/245947
Volume of graphic consultation, (M/F)	246748/197689
Volume of telephone consultation, (M/F)	36909/35713
Volume of video consultation, (M/F)	10063/12545

The platform is a licensed online mental health service provider in China. The data were obtained under a formal research collaboration agreement, which mandated compliance with China’s Personal Information Protection Law (2021) and the platform’s internal data governance policies. The study utilized de-identified administrative data from the Haoxinqing Platform. Prior to delivery, the dataset underwent thorough de-identification, removing all personally identifiable information (PII), including names, contact details, medical license numbers.

### Data screening and processing

From January 2018 to June 2023, a total of 11,333 mental health practitioners registered on the Haoxinqing platform, spanning 16 professional fields, including psychological consultants, nurses, and pharmacists. To align with the study’s focus on professional physicians within China’s medical system, we implemented a rigorous screening process. First, we excluded 244 psychological consultants due to their lack of medical background and focus on counseling or psychotherapy. Second, 14 intern and assistant doctors were excluded as they lack independent prescription rights. Finally, 8 social workers, nurses, and pharmacists were excluded due to their distinct roles from clinical physicians. After screening, 11,067 physicians remained, including resident physicians, attending physicians, associate chief physicians, and chief physicians, all holding valid practicing certificates and central to medical service delivery. These data were used for demographic and medical service correlation analyses.

### Data preprocessing

This study divided the collected data into two major categories: doctors’ demographic characteristics and medical service data.

Demographic characteristics: We summarized information on doctors’ gender, age, practice region, professional title, and hospital level. Hospital levels were classified according to the *Hospital Classification and Management Standards*, categorizing the 2,532 hospitals included in the Haoxinqing platform into Level 1, Level 2, and Level 3. Hospitals that were not graded or were Internet hospitals were classified as “others”.
Medical service data: We counted the number of patients for each doctor, the number of prescriptions, and the number of consultations conducted through three different consultation modes. On the Haoxinqing platform, patients can choose doctors to establish a doctor-patient relationship by directly following the doctors’ personal homepages and select one of the following three consultation methods according to their needs: text and image consultation, phone consultation, and video consultation.

To further analyze physician service patterns, we calculated two metrics: (1) consultation frequency (the ratio of consultations to patients), used to assess physician service activity and patient consultation demand intensity; and (2) prescription intensity (the ratio of prescriptions to consultations), used to measure physician prescription tendencies. By excluding non-consulting patients and non-prescription consultations, these metrics provide a more accurate reflection of physician service characteristics and clinical decision-making patterns.

### Statistical analysis

Descriptive statistical analyses, including bar charts, line charts, and pie charts, were generated using Microsoft Excel to visualize the distribution of physician characteristics and medical service data.

Core statistical analyses were performed using SPSS Statistics. Non-parametric tests were selected due to the non-normal distribution of patient volume and prescription data (Shapiro-Wilk test, *P* < 0.05). The Kruskal-Wallis H test was used to compare patient volume and prescription volume across the four professional title groups, while the Mann-Whitney U test assessed gender differences. The chi-square test examined the association between professional title and gender, as well as the impact of gender and professional title on consultation method selection. Statistical significance was set at *P* < 0.05. Bonferroni correction was applied to address Type I error risks in multiple comparisons, and adjusted significance levels are reported.

To model count-type service data (patient count, prescription count) with overdispersion, negative binomial regression was employed. Independent variables included professional title (categorical), gender (binary), age (continuous), and hospital level (categorical). Results were reported as incidence rate ratios (IRR) with 95% confidence intervals. Model suitability was confirmed via likelihood ratio tests (vs. Poisson regression), and goodness of fit was evaluated using the Akaike Information Criterion (AIC).

## Results

### Demographic distribution characteristics of physicians on the platform

**Figure 1:**
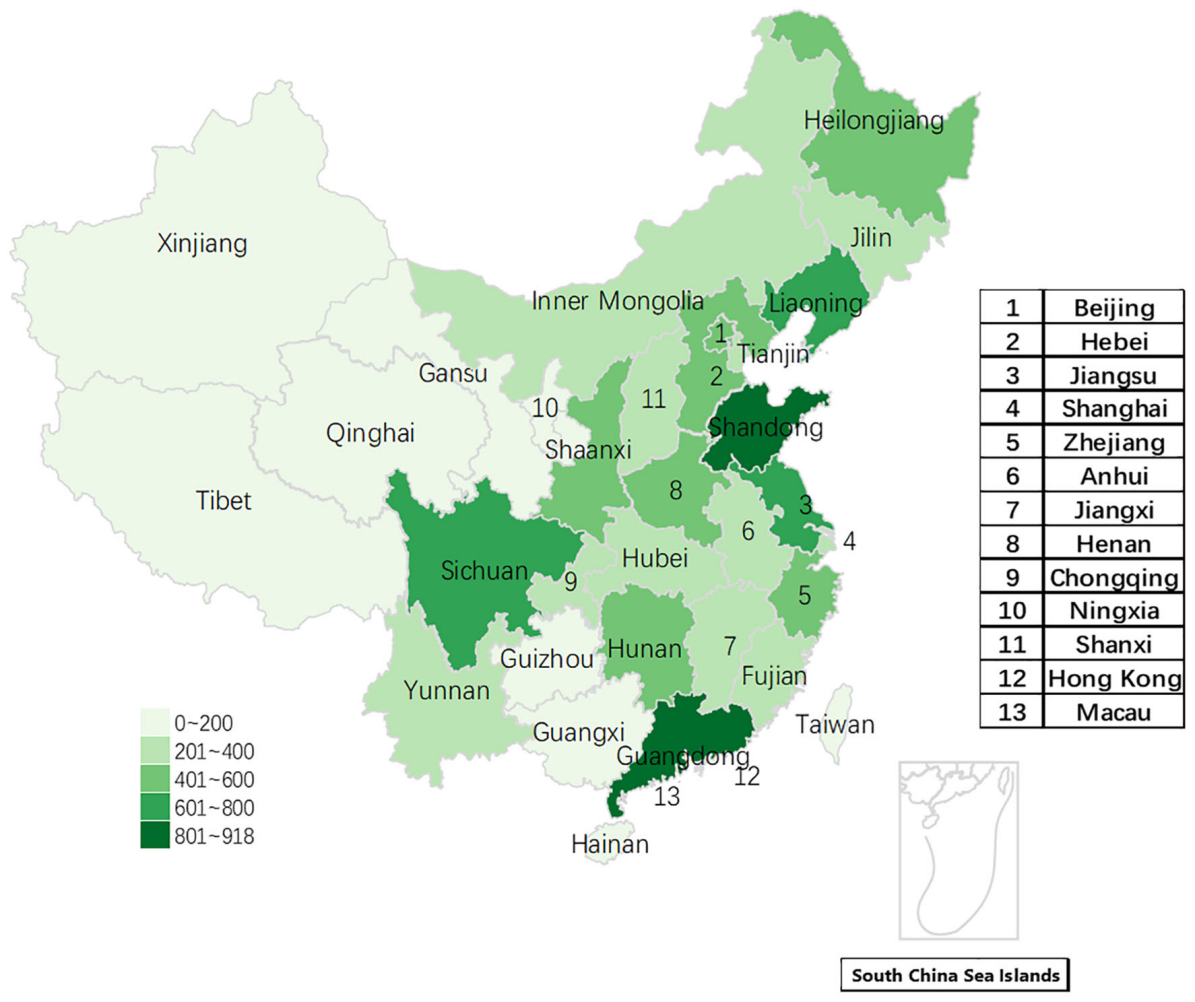
The geographical distribution of registered users on the Haoxinqing platform.

**Figure 1** reveals the distribution of registered doctors on the Haoxinqing platform across different provinces in China. Guangdong Province tops the list with 918 registered doctors nationwide, followed closely by Shandong Province and Jiangsu Province, with 808 and 797 registered doctors respectively. In contrast, with the exception of Sichuan Province (634 registered doctors, ranking fifth), the number of registered doctors in the western region provinces is significantly lower than that in the coastal provinces of the eastern region.

As shown in **Table 2**, among the senior titles of chief physician and associate chief physician, the number of male physicians is slightly higher than that of female physicians. In contrast, in the middle and lower titles, such as resident physician and attending physician, the number of registered female physicians shows a slight predominance. Specifically, among attending physicians, there are 2117 female physicians compared to 2073 male physicians. In the resident physician title, the number of female physicians is 809, exceeding the 674 male physicians. The chi-square analysis results show a significant association between gender and professional title (*χ* ^2^ = 29.285, *P* < 0.001, Cramer’s *V* = 0.051), indicating a weak effect size (Cramer’s *V*<0.1). Additionally, the data indicate that the platform has a higher number of attending physicians and associate chief physicians, who together account for 65.5% (7246/11067) of all registered physicians.

**Table 2:** The results of the chi-square analysis of the distribution of professional titles.

Category	Male	Female	*χ* ^2^	*P*
Attending physician	674 (12.1%)	809 (14.8%)	29.285	<0.001
Attending Physician	2073 (37.1%)	2117 (38.7%)		
Associate Chief Physician	1729 (30.9%)	1507 (27.5%)		
Chief Physician	1116(20.0%)	1042(19.0%)		

Cramer’s V=0.051, P-values adjusted via Bonferroni correction for multiple comparisons.

**Figure 2:**
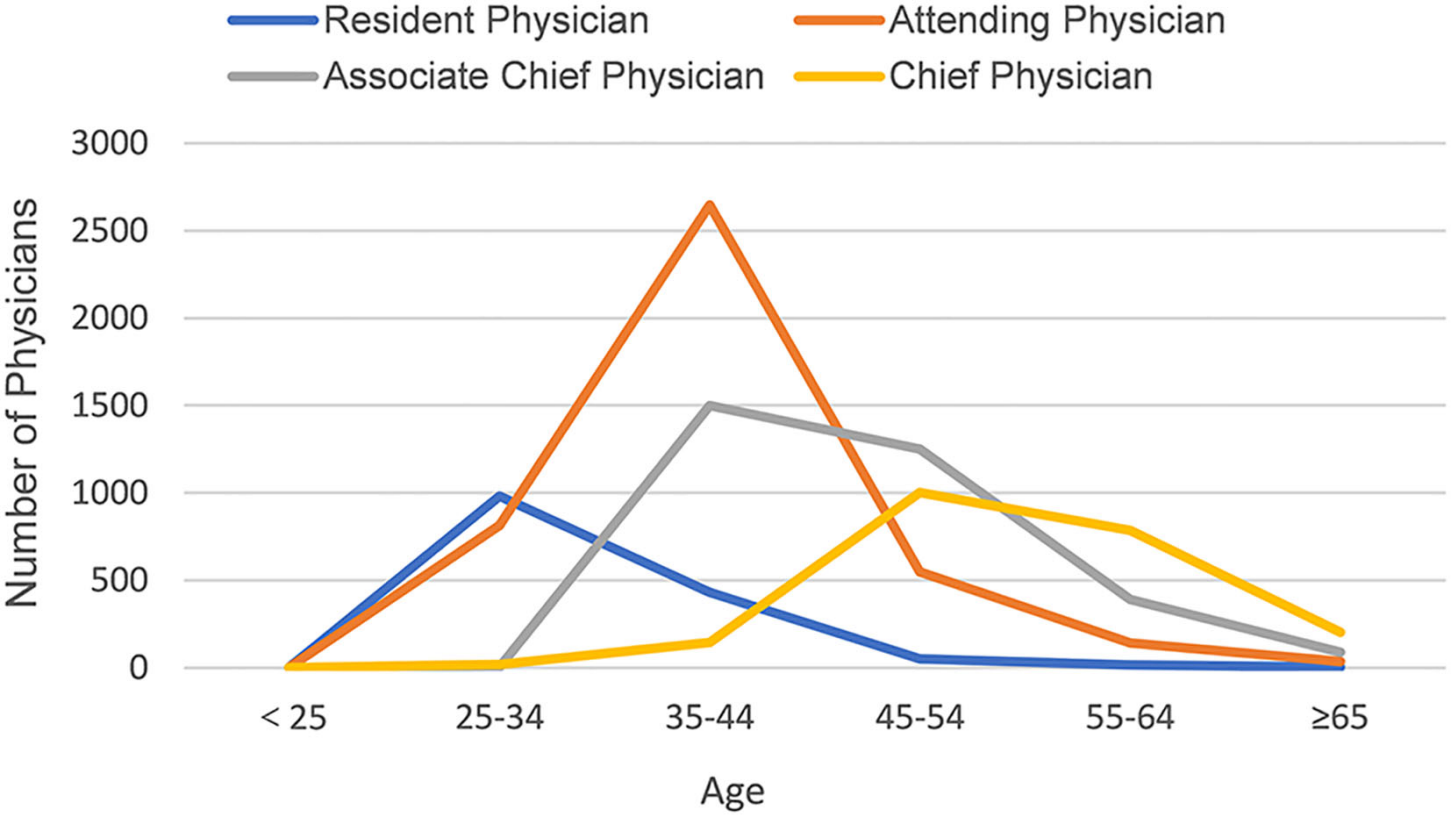
The age distribution of doctors with different professional titles.

As shown in **Figure 2**, the age distribution of resident physicians and attending physicians shows a single peak, reaching the peak in the age groups of 25–34 years and 35–44 years, respectively. In contrast, the age distribution of associate chief physicians and chief physicians shows a bimodal pattern. Associate chief physicians reach peaks in the age groups of 35–44 years and 45–54 years, while chief physicians are primarily concentrated in the age groups of 45–54 years and 55–64 years.

**Figure 3:**
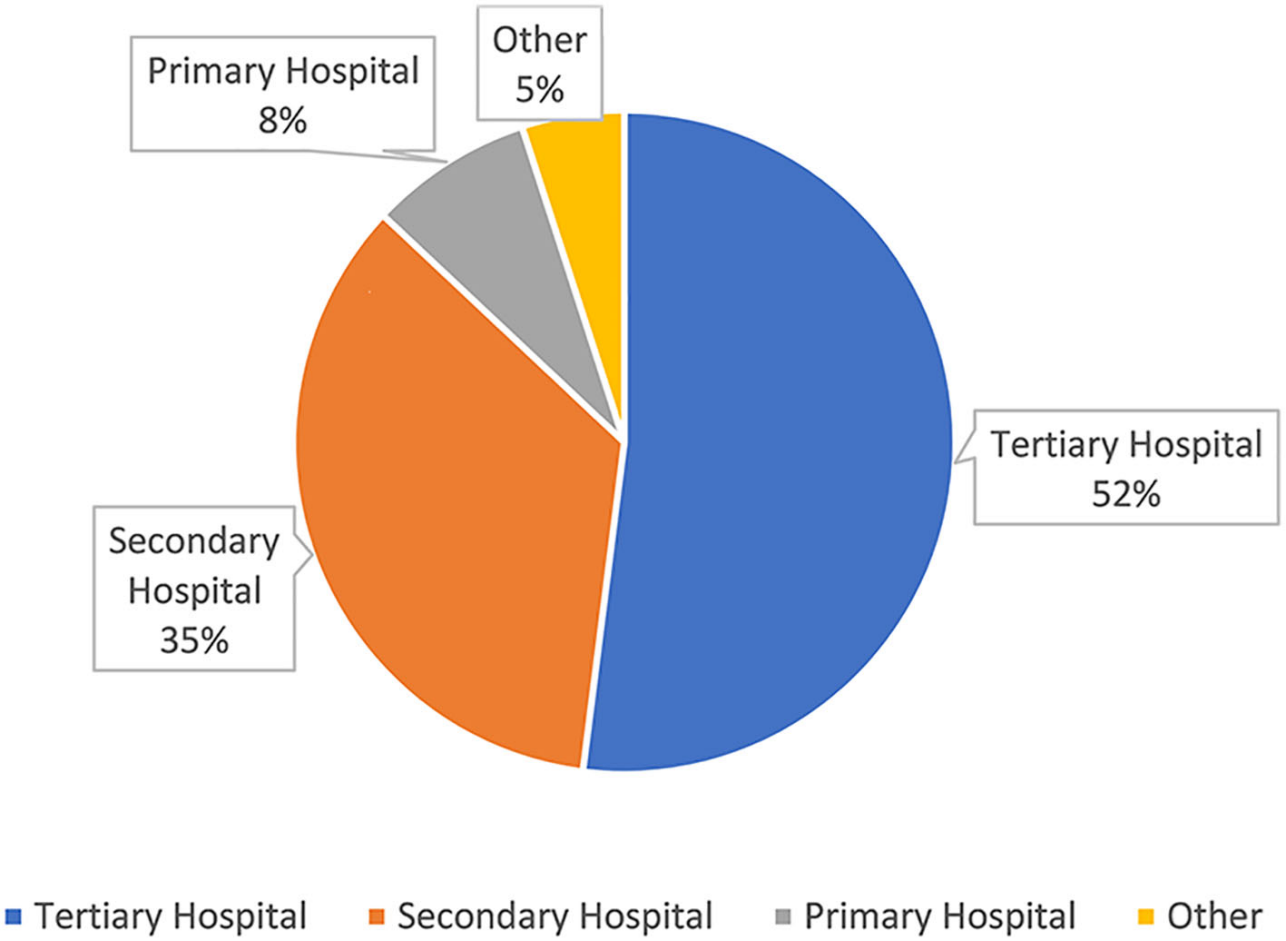
The distribution map of the hospital levels of Haoxinqing.

**Figure 3** displays the distribution of the levels of medical institutions to which the physicians on the platform belong. It can be observed that tertiary hospitals account for the highest proportion at 52%, followed by secondary hospitals at 35%, while primary hospitals and other non - graded hospitals only account for 13%.

### Characteristics of medical service data

**Figure 4:**
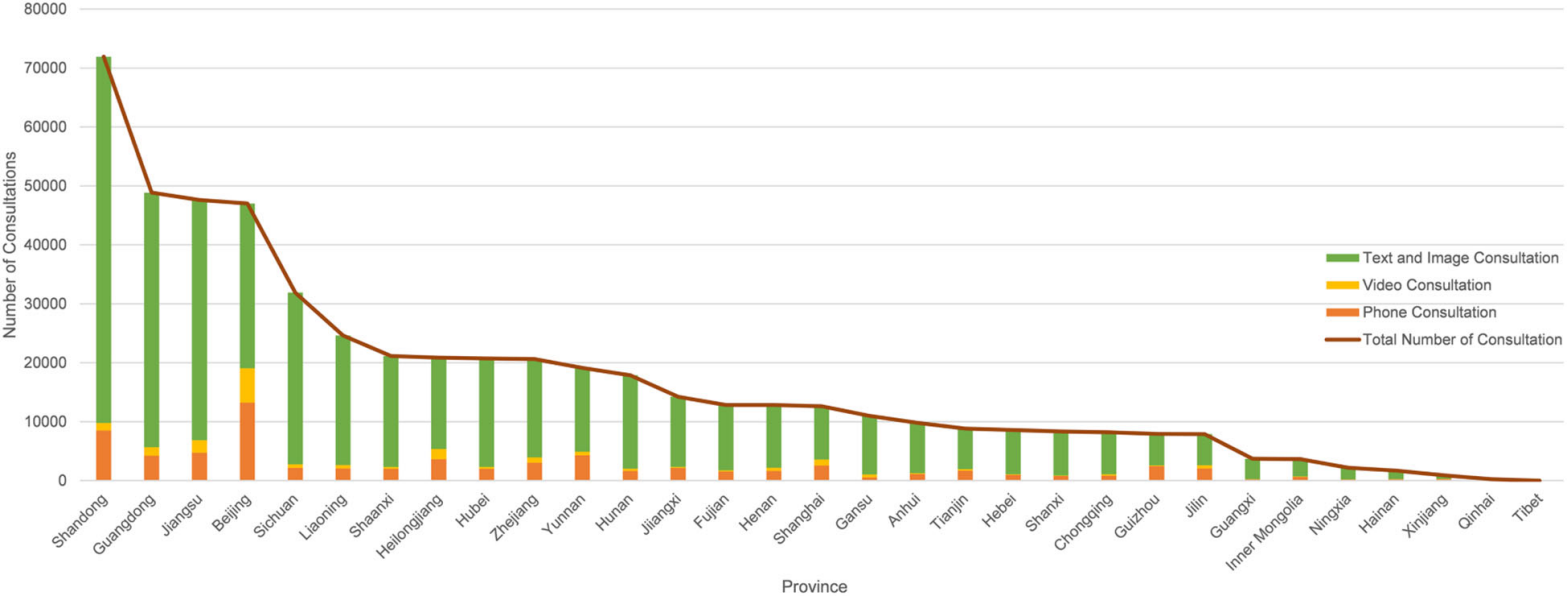
Distribution map of consultation methods in each province.

When analyzing the distribution of consultation volumes and methods on the Haoxinqing platform across different regions in China, as shown in **Figure 4**, Shandong Province ranks first with 71,916 consultations, accounting for 13.6% of the total consultations. Guangdong Province follows with 48,848 consultations (9.2%), and Jiangsu Province with 47,600 consultations (9.0%). Notably, among the 31 provinces included in the statistics, the sum of consultations from the top 15 provinces accounts for 81.9% of the national total (432,103/527,645). Furthermore, an analysis of the distribution of consultation methods across provinces reveals that text and image consultations dominate in all provinces, with an average share of 82.1%. However, in Beijing, phone and video consultations account for 40.6% of the total consultations, a relatively high proportion compared to other provinces.

**Figure 5:**
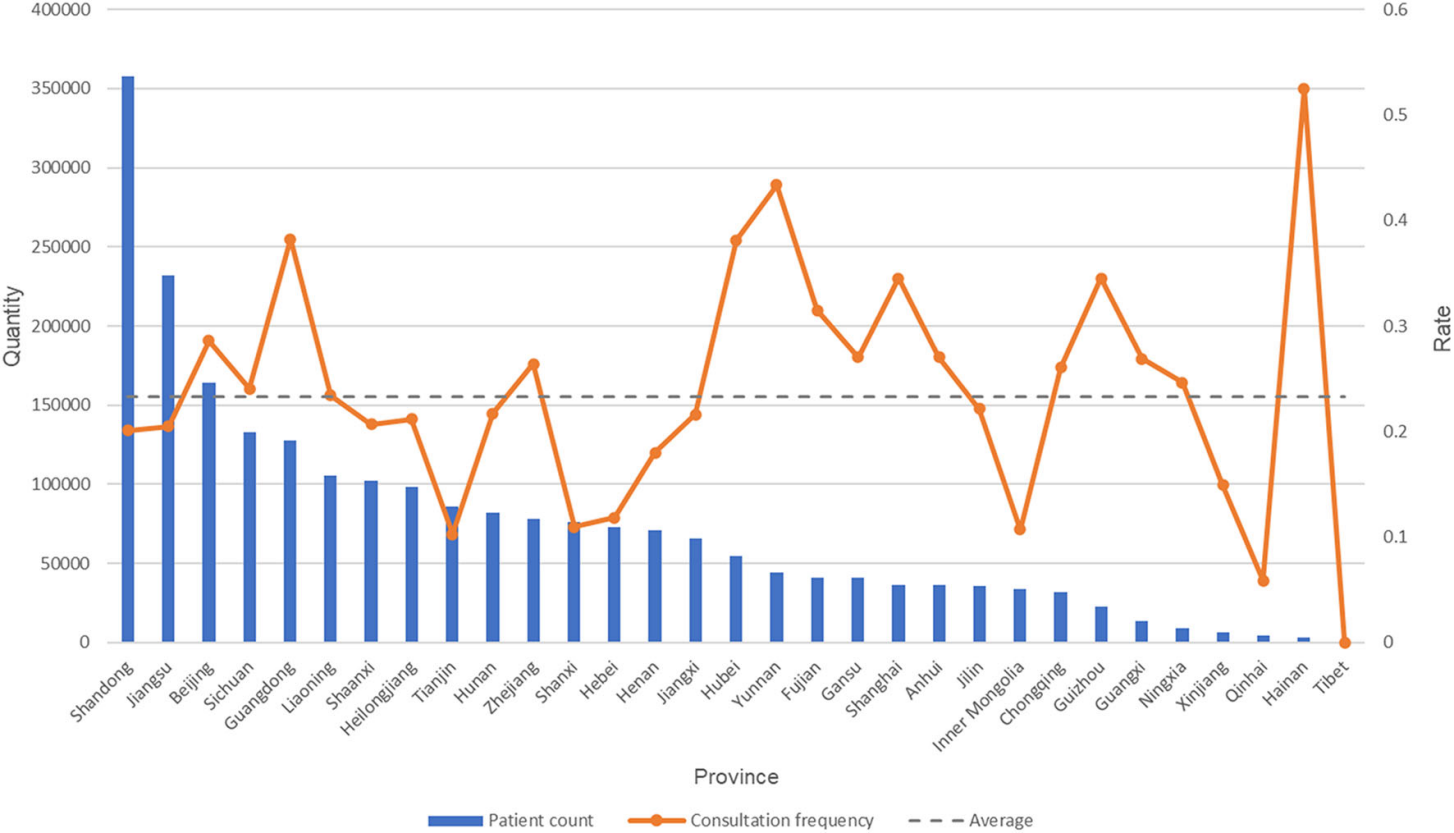
The number of patients and the consultation conversion rate in each province.

**Figure 5** further analyzes the service conditions of each province using the consultation frequency we defined. The line chart in the figure reflects the consultation frequency of patients in each province. It can be observed that, despite having the largest number of patients, both Shandong Province and Jiangsu Province have consultation frequencies lower than the national average. In contrast, Beijing and Guangdong Province not only have a large number of patients but also rank among the top in consultation frequency. In addition, some provinces with relatively fewer patients show significant consultation frequencies. For example, Hainan Province, which ranks 30th in the number of patients, has the highest consultation frequency in the country; Yunnan Province, ranking 17th in the number of patients, has the second-highest consultation frequency nationwide.

As shown in **Supplementary Table 1** (**Supplementary Material**), there are significant differences in the service models of doctors with different professional titles. The prescription intensity of attending physicians is the highest (3.55), significantly higher than that of resident physicians (2.15) and senior title doctors (associate chief physician: 3.17; chief physician: 3.07). The consultation frequency is the highest among associate chief physicians (0.263).

### The relationship between physicians’ demographic data and medical service data

**Figure 6:**
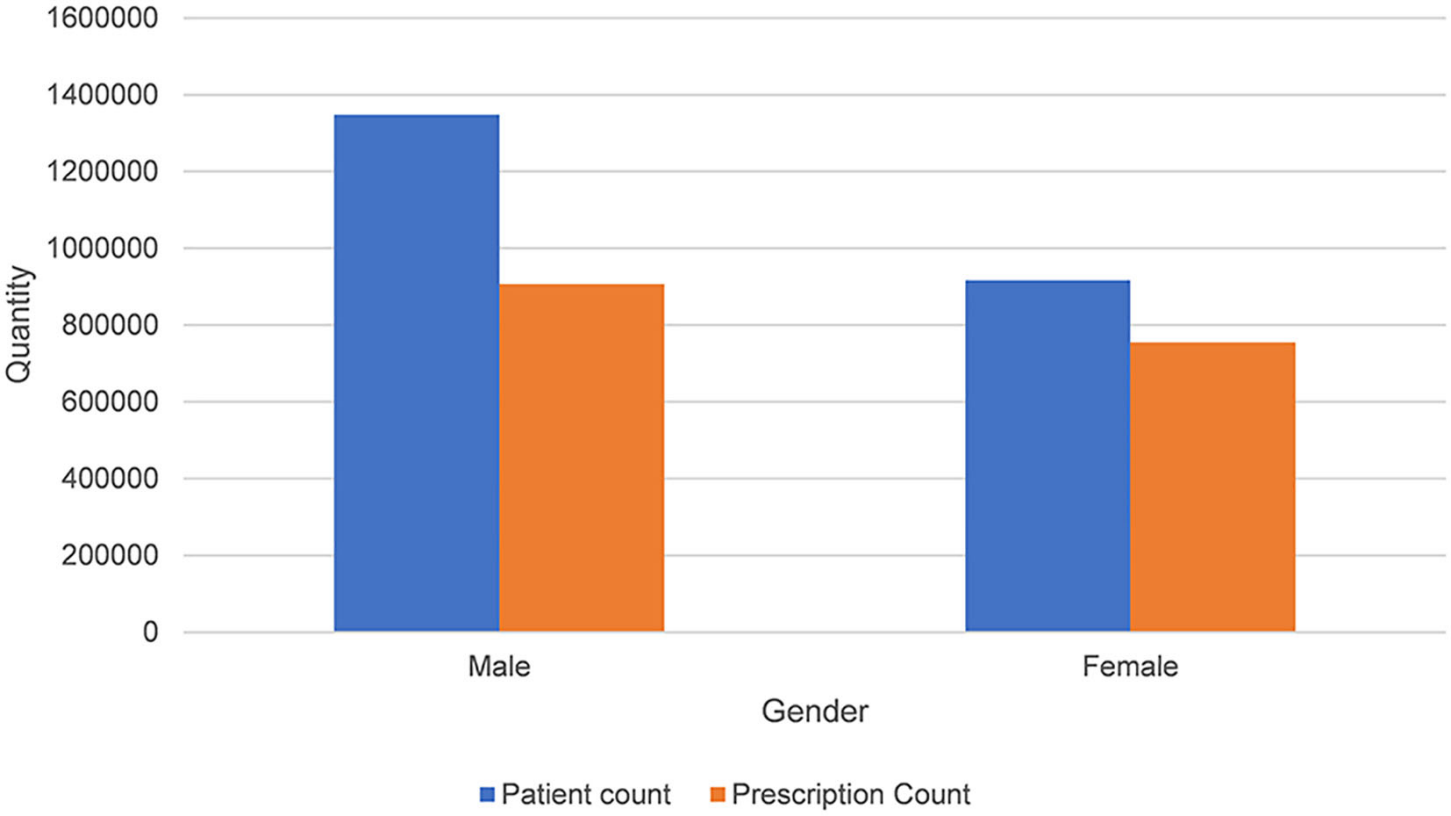
Distribution of patient volume and prescription volume by gender.

As illustrated in **Figure 6**, male physicians on the Haoxinqing platform demonstrated higher patient and prescription volumes compared to their female counterparts. However, **Supplementary Table 2** further analysis using the Mann-Whitney U test indicated no statistically significant differences between genders in terms of patient counts (*U*=0.715, *P*=0.475, effect size *r*=0.004) or prescription counts (*U*=0.994, *P*=0.320, effect size *r*=0.003), with negligible effect sizes (*r*<0.1).

As shown in **Supplementary Table 3**, the chi-square analysis shows a significant association between physician gender and consultation methods (*χ* ^2^ = 677.555, *P* < 0.001, Cramer’s *V*=0.036). Text and image consultation accounted for 83.7% of the total consultations of male physicians, slightly higher than that of female physicians (81.2%). In contrast, female physicians had slightly higher proportions in telephone consultation (14.3% vs. 12.9%) and video consultation (4.5% vs. 3.4%) compared to male physicians.

The results of the Kruskal-Wallis H test examining the relationship between physician rank and both patient volume and prescription volume are presented in **Supplementary Table 4**. The analysis revealed significant differences in patient counts across physician ranks (*H* = 565.098, *P* < 0.001, *η*²=0.051). Resident physicians had a median patient count of 12 (IQR: 4, 45), which increased to 22 (6, 81) for attending physicians, 39 (9, 132) for associate chief physicians, and 60 (13, 216) for chief physicians. Similarly, significant differences were observed in prescription counts (*H* = 360.338, *P* < 0.001, *η*²=0.032). Resident physicians had a median prescription count of 7 (IQR: 2, 39), which rose to 15 (2, 79) for attending physicians, 29 (4, 134) for associate chief physicians, and 41 (4, 203) for chief physicians.

As shown in **Supplementary Table 5**, the chi-square analysis revealed significant associations between physician rank and consultation methods (*χ* ^2^ = 44633.592, *P* < 0.001, Cramer’s *V*=0.065). Resident physicians predominantly utilized image-text consultations (90.1%), with lower proportions for phone (8.1%) and video consultations (1.8%). Attending physicians showed a similar pattern, with 84.8% image-text consultations, 11.8% phone consultations, and 3.4% video consultations.

For associate chief physicians, 84.3% of consultations were image-text, 12.4% were phone, and 3.3% were video. Chief physicians had a slightly lower proportion of image-text consultations (78.6%), with higher rates of phone (16.3%) and video consultations (5.1%).

Negative binomial regression analysis (**Table 3**) revealed that:

Prescription Count: Negative binomial regression revealed significant associations with professional titles: Chief physicians prescribed 4.16 times more than residents (95% CI [3.21–5.41], *P* < 0.001), followed by associate chiefs (Exp(*B*) = 2.66) and attendings (Exp(*B*) = 1.82). Male physicians prescribed marginally less than females (Exp(*B*) = 0.89, *P* = 0.047), while age showed minimal positive effects (Exp(*B*) = 1.01, *P* = 0.001). Hospital level had no impact (*P* ≥ 0.096).
Patient Volumes: Higher titles predicted greater patient count: Chiefs attracted 3.85 times more patients than residents (95% CI [2.11–7.00], *P* < 0.001), with associate chiefs (Exp(*B*) = 2.02) and attendings (Exp(*B*) = 1.38) following. Male physicians had 30% fewer patients than females (Exp(*B*) = 0.70, *P* < 0.001). Age and hospital level were non-significant (*P* ≥ 0.162).

**Table 3:** Negative binomial regression analysis of factors associated with prescription count and patient volume.

Variable	Prescription Count		Patient Count	
	Exp(*B*) [95% Cl]	*P*–value	Exp(*B*) [95% Cl]	*P*–value
Intercept	19.78 [8.37ception	<0.001	207.70 [69.04eption,	<0.001
Professional Title (Ref: Resident)				
Chief	4.16 [3.21t)eona	<0.001	3.85 [2.111)eona	<0.001
Associate Chief	2.66 [2.16iatena	<0.001	2.02 [1.211atena	0.008
Attending	1.82 [1.54dingna	<0.001	1.38 [0.871ingna	0.173
Gender (Ref: Female)				
Male	0.89 [0.80 Fegna	0.047	0.70 [0.58 Fegna	<0.001
Age	1.01 [1.011Fegna	0.001	0.99 [0.981Fegna	0.276
Hospital Level (Ref: Primary)				
Other	0.96 [0.41)Prgna	0.918	2.66 [0.68)Prgnal	0.162
Tertiary hospital	1.96 [0.89talgna	0.096	0.81 [0.33talgna	0.632
Secondary hospital	1.02 [0.46talyna	0.969	0.74 [0.30talyna	0.522

## Discussion

### Principal findings

A total of 87% of registered physicians are affiliated with secondary or tertiary hospitals, predominantly located in eastern coastal provinces such as Guangdong and Shandong. While developed provinces have the highest consultation volumes, consultation frequency peaks in resource-scarce regions like Hainan.

Gender is significantly associated with professional title distribution (*χ*²=29.285, *P* < 0.001), but the effect size is minimal (Cramer’s *V*=0.051), indicating limited practical differences. Similarly, although gender significantly influences consultation modality choices (*χ*²=677.555, *P* < 0.001), the effect size remains small (Cramer’s *V*=0.036). These low effect sizes suggest that despite observable gender-related patterns in the platform data, their actual clinical or managerial significance may be limited and require further interpretation in the context of social role theory.

Professional titles significantly predict service volume differences: chief physicians have 3.85 times the patient volume (Exp(*B*)=3.85, 95% CI [2.11–7.00], *P* < 0.001) and 4.16 times the prescription volume (Exp(*B*)=4.16, [3.21–5.41], *P* < 0.001) of residents, with associate chiefs and attendings showing a stepwise decrease.

Nationally, text and image consultations dominate (82.1%), but Beijing demonstrates a unique adoption of phone/video consultations (40.6%), likely driven by high-income demographics and provider competition.

### Challenges and countermeasures of mental health service resource allocation under the “Matthew effect”

The data shows that the number of doctors registered on the Haoxinqing platform is highest in the economically developed provinces of Guangdong, Shandong, and Jiangsu, and that doctor registration is mainly concentrated in the eastern coastal provinces. This is consistent with the findings of a cross-sectional study on Internet hospitals by Li et al, which also reported that the number of registered physicians was primarily concentrated in eastern regions (17). This concentration trend is attributed to policy bias. Taking Guangdong Province as an example, as a pioneer in the development of internet hospitals, Guangdong was the first to allow medical institutions to use “internet hospital” as a secondary name and achieved full coverage of internet medical construction in all provincial tertiary hospitals (22, 23).

However, this policy bias and technological agglomeration further strengthen the resource advantages of core areas, creating a “Matthew effect” in internet-based medical care (24). That is, high-quality online medical resources are concentrated on leading platforms, enabling top-tier hospitals to attract patients from across the country through their brand influence and thus further consolidating their core positions. The Haoxinqing platform is no exception. Data indicates that institutions of secondary level and above account for 87% of the platform, which is also related to China’s three-tier medical system: primary-level institutions handle common diseases and health management; secondary-level institutions receive referrals and deal with complex diseases; and tertiary-level institutions diagnose and treat difficult and critical cases and undertake scientific research and teaching (25). Regrettably, the hierarchical diagnosis and treatment in the field of mental health has not yet matured. Specialized resources are still concentrated in developed cities, while primary-level service capabilities are weak. This forces patients to preferentially choose municipal specialized hospitals or the psychological departments of tertiary hospitals (26).

It is worth noting that the consultation frequency in physician resource-rich provinces such as Shandong and Jiangsu are lower than the national average. In contrast, the high consultation frequency in resource-scarce regions such as Hainan (>50%) precisely reflects the compensatory role of internet-based medical care for offline services (27). Developed provinces are early adopters in Rogers’ framework, where favorable policy environments accelerate innovation assimilation. Resource-scarce regions represent the late majority adopters, where deficiencies in traditional medical systems amplify the “relative advantage” of internet-based healthcare.

The rise of internet hospitals has provided dual possibilities for mental health services. On the one hand, patients can access high-quality resources across regions, alleviating the medical difficulties in resource-scarce areas. On the other hand, the distribution of online medical resources is still deeply embedded in the traditional hierarchical system, forming a “Matthew effect.” Future policies can focus on the following two aspects: First, establish a “digital reverse nurturing” mechanism. For example, stipulate that core hospitals allocate a certain proportion of online service quotas to resource-scarce areas to curb excessive resource concentration. Second, strengthen the information capacity building of primary-level medical institutions, support the internet medical construction of primary hospitals, and promote the in-depth integration of hierarchical diagnosis and treatment with internet hospitals.

### Weak effects, persistent gaps: decoding systemic gender disparities in medical hierarchies through effect size and social role lenses

Despite achieving near-gender parity among registered physicians (male: female = 5659: 5674), significant disparities were observed in professional title distribution: women accounted for 38.7% of attending physicians but only 19.0% of chief physicians (*χ*² = 29.285, *P* < 0.001; Cramer’s *V* = 0.051). Previous studies have demonstrated that stereotypical gender roles influence the overall career planning and transitions of female physicians (28). However, the weak effect size (*V* < 0.1) suggests that the association may be substantively negligible. Social role theory can explain this “significant yet weak” paradox through the following mechanisms: the resistance of young female physicians to traditional gender roles (e.g., delaying childbirth) partially offsets the impact of societal expectations. For instance, a study from Hungary revealed that female physicians face a “dual role expectation” (professional competence and family responsibilities) and may passively delay childbirth due to concerns about career advancement (29). On the other hand, the population of registered physicians on the platform may have filtered out women who left due to role conflicts, resulting in observed gender differences that are smaller than those in the real workplace and thus lower effect sizes. Previous research has also shown that gender differences in physician promotion persist even after controlling for childbirth, number of children, and productivity, indicating that the career development system is more favorable to men (30). Therefore, the weak statistical association does not negate the necessity for intervention. Eliminating occupational bias requires transcending statistical debates and focusing on institutional reconstruction and innovation in role expectations.

The research data indicate that, although nonparametric tests showed no significant differences between male and female physicians in prescription volume (*P*=0.320) and number of patients (*P*=0.475), negative binomial regression models suggested that male physicians had significantly fewer patients than female physicians (Exp(*B*) = 0.70, *P* < 0.001), indicating that, after controlling for professional title, age, and hospital level, female physicians saw more patients. This may be because negative binomial regression, by controlling variables such as professional title, revealed gender effects masked by the original data. Chi-square analysis showed a significant association between physician gender and consultation methods (*χ*² =677.555, *P* < 0.001). Specifically, female physicians had a higher proportion of telephone and video consultations, which is consistent with the social role theory that female physicians, due to societal expectations of them as “caretakers”, may prefer consultation methods that involve higher emotional investment. These methods require stronger empathetic abilities and time commitment, aligning with the stereotype of women as “patient and meticulous”. Similar to previous studies, female physicians are generally better at listening to patients’ needs and feelings, tend to spend more time communicating with patients, and adopt a more patient-centered communication style (31), investing more time in each consultation (32). However, the chi-square analysis results showed a low effect size, Cramer’s *V* = 0.036, indicating a weak association strength. Our analysis found that, regardless of gender, text-based consultation dominated on the platform (>80%), suggesting that the overall service design of the platform leans toward an efficient and quick mode, which may suppress the manifestation of gender differences. However, a recent study indicated that women are expected to possess both “professional competence” and “emotional care”. Patients are more sensitive to the technical abilities of female physicians, while being more lenient in their evaluations of male physicians. For example, the same technical mistake may label a female physician as “incompetent”, while a male physician may be attributed to a “random error” (33).

To resolve the conflict between gender role expectations and clinical practice, a multifaceted performance evaluation system should be established, incorporating quality indicators such as patient satisfaction and health outcomes. The intelligent triage system should be optimized to match the strengths of female physicians in emotional care. Additionally, an anonymous evaluation mechanism should be implemented to reduce patient bias, and data transparency should be enhanced through robust regulatory oversight. These measures can systematically dismantle the dual constraints of “competence and care”, transforming female physicians’ communication strengths into career capital and promoting substantive equity and service quality improvement within the healthcare system.

### Professional titles and age distribution: the current status of career advancement for doctors

Characteristics of the distribution of titles and ages on the Haoxinqing platform: Young doctors (aged 25–34) are predominantly concentrated in lower-ranking positions such as resident physicians and attending physicians, while older doctors (aged 45–54) are more likely to hold higher-ranking positions such as associate chief physicians and chief physicians. This pattern is consistent with China’s existing title promotion system, which stipulates the minimum years of service required for promotion to higher-level titles. For instance, physicians with a bachelor’s degree must have at least 15 years of work experience to be eligible for promotion to chief physician (34).

The current title promotion system has significant issues. Chinese physicians generally express low overall satisfaction with the title promotion process. First, it relies excessively on administrative evaluation and lacks a scientific and systematic job evaluation system, resulting in evaluation content that lacks job specificity and scientific rigor. Second, the current salary system, which is primarily based on title, education level, and years of service, fails to adequately reflect the clinical capabilities and work performance of medical staff. Moreover, primary care physicians face promotion barriers due to the high demands for research and publications, which are not aligned with their clinical practice-oriented job nature (35). Therefore, the reform of the title promotion system should balance clinical practice and research capabilities, establish tiered and categorized promotion criteria, and formulate differentiated requirements based on regional medical needs and hospital functional positioning. In particular, the promotion conditions for primary care physicians should be relaxed, and more support and development opportunities should be provided to promote the rational allocation of medical resources and coordinated development of the industry (36).

### Impact of professional title on medical service data

The study results show that professional title has a significant impact on medical service data. There are significant differences in prescription volume, patient volume, and consultation method preferences. Specifically, physicians with higher professional titles have significantly higher patient numbers and prescription numbers than those with lower titles, and patients are more inclined to choose higher title physicians for video and telephone consultations. This finding is consistent with the results of Li et al. (17), which may be because higher title physicians usually have richer clinical experience and higher professional technical levels, often undertaking more complex diagnostic and treatment tasks, and thus are more likely to gain patient trust, making patients more willing to choose the more expensive telephone and video consultation methods over text and image consultations. The study by Sun et al. further explains how professional titles influence patient trust: professional titles indirectly affect physicians’ psychological resilience through their associations with age, income, and job stress, which in turn impacts patient trust. Specifically, senior physicians may gain greater patient trust due to the accumulation of clinical experience and higher income (37). Furthermore, the strong correlation between professional titles and online reputation (e.g., review ratings, patient-generated content) creates a self-reinforcing cycle: higher-titled physicians attract more patients through algorithmic prioritization, which in turn amplifies their visibility and perceived authority (38).

Lastly, it is noteworthy that Beijing exhibits a unique service pattern. Despite not ranking in the top five in terms of the number of registered physicians, it has the highest proportion of telephone/video consultations at 40.6%, which is significantly higher than that of other provinces. This discrepancy likely stems from two main factors. First, as the capital, Beijing is home to a large number of high-income and highly educated individuals who place a greater emphasis on privacy and in-depth communication, thus favoring video consultations that closely resemble offline clinical interactions. Second, the high density of tertiary hospitals in Beijing drives physicians to differentiate their services (e.g., by offering video consultations) to avoid the homogenization risks inherent in text/image modalities. This highlights that even in resource-rich areas, the synergy between policy and patient needs can foster service innovation. Optimizing resource allocation through regional pilots (e.g., expanding the scope of insurance coverage for telehealth) may be more effective than mere quantitative expansion of resources.

### Limitations

This study has several limitations that should be taken into account. First, the dataset mainly focuses on mental health professionals registered on the platform while lacking detailed patient-level data, including clinical diagnoses, demographic characteristics, and satisfaction metrics. The absence of multidimensional patient profiles to some extent limits the description of the personalized features of digital mental health platforms. Second, due to the inherent measurement limitations of retrospective service data, the operational definitions of consultation frequency and prescription intensity may not be able to fully capture patient engagement and doctors’ prescription tendencies, and there may be measurement bias. In addition, as a pioneering cross-sectional study of internet hospital applications in China’s mental health field, the lack of comparable benchmark data from previous studies means that the research findings need to be interpreted with caution. We acknowledge that the sample represents voluntary platform users and may over-represent tech-savvy physicians in developed areas, limiting generalizability to offline clinical settings or resource-scarce regions. Future studies should collect patient feedback and clinical data to more comprehensively evaluate the performance of internet hospitals dedicated to mental health services.

## Conclusion

This study, based on cross-sectional data from China’s largest online mental health platform, integrates the theories of diffusion of innovation and social roles to uncover the associations between physician demographics and online service behaviors. The findings indicate that while online services complement the offline system, their essence remains embedded in traditional medical hierarchies, exemplifying a “system replication empowered by technology”. The study finds that patients’ preferential trust in senior physicians and gendered communication norms in medical services are important factors influencing the differentiated service patterns of resource allocation on digital platforms. Future research should integrate patient-end data to break through the “Matthew effect” in resource allocation, promote counterintuitive resource distribution and reform of title evaluation systems, and drive the transformation of digital healthcare from a technological tool to a systemic restructuring.

## Data Availability

The data contain sensitive information about mental health professionals and patients, and their use is governed by strict privacy regulations. Requests to access these datasets should be directed to 47414030@qq.com.
