# Supplementary material for: Analysis of psychiatrists’ internet service patterns: a cross-sectional study from China’s largest online mental health platform
Source: Front Psychiatry. 2025 Jun 26;16:1598574. doi: 10.3389/fpsyt.2025.1598574 (PMC12241070; doi:10.3389/fpsyt.2025.1598574)
Supplement: Supplementary file 1 [file Table1.docx]

Supplementary Table 1: Prescription intensity and consultation frequency of doctors with different professional titles

| Category | Prescription Intensity | Consultation Frequency |
| --- | --- | --- |
| Resident Physician | 2.15 | 0.203 |
| Attending Physician | 3.55 | 0.182 |
| Associate Chief Physician | 3.17 | 0.263 |
| Chief Physician | 3.07 | 0.254 |

Supplementary Table 2: The results of the Mann-Whitney U test on the relationship between physician gender and the number of prescriptions and patients.

| Category | Male (*N*=5592) | Female (*N*=5475) | *Z* | *P* |
| --- | --- | --- | --- | --- |
| Patient Count | 29(7,114) | 29(8,106) | 0.715 | 0.475 |
| Prescription Count | 19(2,109) | 20(3,105) | 0.994 | 0.320 |

Note: Both effect sizes are negligible. *P*-values adjusted via Bonferroni correction for multiple comparisons.

Supplementary Table 3: The results of the chi-square analysis on the relationship between physician gender and consultation methods.

| Category | Male | Female | *χ*^2^ | *P* |
| --- | --- | --- | --- | --- |
| Text and Image Consultation | 238684(83.7%) | 196862(81.2) | 677.555 | <0.001 |
| Phone Consultation | 36656(12.9%) | 34712(14.3%) |  |  |
| Video Consultation | 9823(3.4%) | 10908(4.5%) |  |  |

Note: Cramer’s *V*=0.036, *P*-values adjusted via Bonferroni correction for multiple comparisons.

Supplementary Table 4: Results of the Kruskal-Wallis H test examining the relationship between physician rank and patient volume, as well as prescription volume.

| Category | Patient Count | Prescription Count |
| --- | --- | --- |
| Resident Physician | 12(4,45) | 7(2,39) |
| Attending Physician | 22(6,81) | 15(2,79) |
| Associate Chief Physician | 39(9,132) | 29(4,134) |
| Chief Physician | 60(13,216) | 41(4,203) |
| *H* | 565.098 | 360.338 |
| *P* | <0.001 | <0.001 |
| Effect Size (*η*²) | 0.051 | 0.032 |

Supplementary Table 5: Results of the chi-square analysis examining the association between physician rank and consultation methods.

| Category | Image-Text Consultation | Phone Consultations | Video Consultations | *χ*^2^ | *P* |
| --- | --- | --- | --- | --- | --- |
| Resident Physician | 29952(90.1%) | 2685(8.1%) | 604(1.8%) | 4463.359 | <0.001 |
| Attending Physician | 98012(84.8%) | 13599(11.8%) | 3946(3.4%) |  |  |
| Associate Chief Physician | 144027(84.3%) | 21136(12.4) | 5601(3.3%) |  |  |
| Chief Physician | 163555(78.6%) | 33948(16.3%) | 10580(5.1%) |  |  |

Note: Cramer's *V*=0.065, *P*-values adjusted via Bonferroni correction for multiple comparisons.
